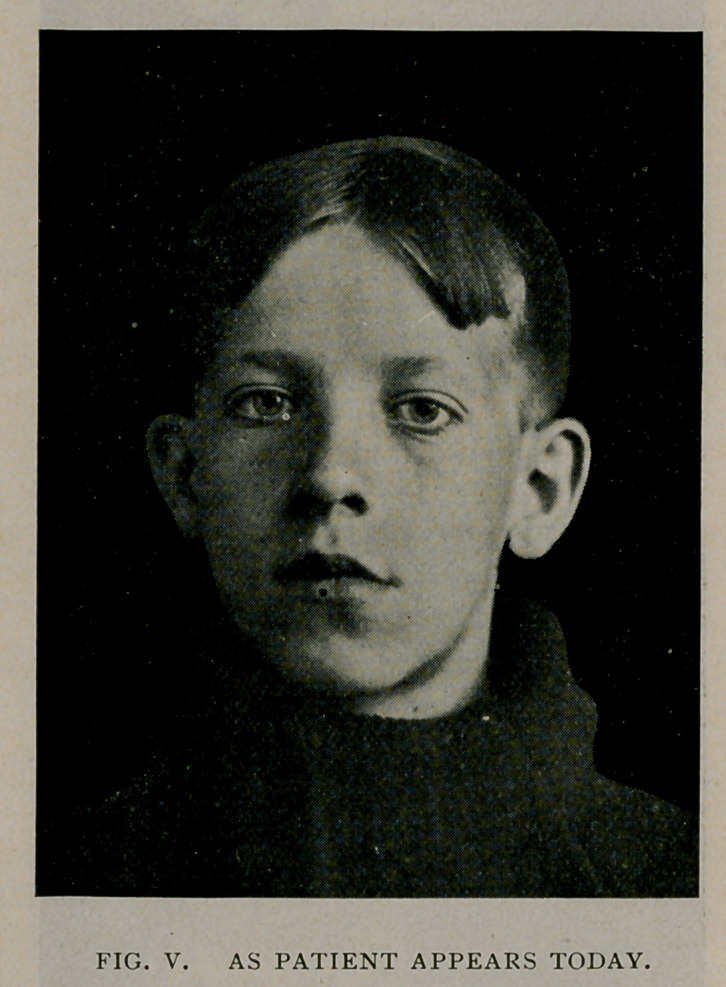# A Case of Multiple Fracture of the Inferior Maxilla Complicated by Dislocation1Read at the annual meeting of the Chautauqua County Medical Society, July, 1900.

**Published:** 1900-10

**Authors:** William Seaman Bainbridge

**Affiliations:** Attending Surgeon Randall’s Island Hospital, Instructor in Operative Surgery New York Post Graduate Hospital Medical College


### A CASE OF MULTIPLE FRACTURE OF THE INFERIOR MAXILLA COMPLICATED BY DISLOCATION.[1]

By WILLIAM SEAMAN BAINBRIDGE, A. M., M. D.,

Attending Surgeon Randall's Island Hospital, Instructor in Operative Surgery New York Post Graduate Hospital Medical College.

MULTIPLE fractures of the lower jaw are deservedly considered by surgeons and surgical dentists as very difficult cases. Reduction under an anesthetic may be easy, but retention in a proper position is very difficult, and the result often far from perfect. I have considered the following case worthy of report because of its complexity and, happily, its satisfactory result.

In August, 1897, I was called with Dr. Jay W. Seaver, of New Haven, Conn., who was associated with me throughout this case, to attend a boy of nine years, who had a few minutes before sustained a severe injury while riding in a hotel elevator. Curious as to the workings of the "lift" our patient,in an effort to see under the elevator, had, while it was in motion, leaned out of the doorway, and his head was caught under the transom of one of the floor doors. Fortunately, the elevator was instantly stopped or a decapitation would have resulted. As it was, the fractures, shown in the accompanying diagram, were sustained.

Besides these, there was a complete loosening of all the incisors in both jaws, and one lower right incisor was extracted at the time of the traumatism. There was considerable laceration of the soft parts especially about the alveolar processes, and the separation of the symphysis was compound. A dislocation of the right temporo-maxillary articulation, which presented marked deformity, added

1. Read at the annual meeting of the Chautauqua County Medical Society, July, 1900.

materially to the difficulty of the case. Marked shock and concussion precluded immediate treatment of the fractures. After the lapse of several hours, reaction set in, chloroform was administered, reduction was made, so far as the marked swelling of all the soft parts permitted, and a combined Barton and Capeline bandage was applied. A small dose of morphine with atropin was given hypodermatically both to quiet the patient and also to lessen the chance of emesis.

During the following five days the patient was fairly comfortable and was fed by means of a small rubber tube passed through the space left by the absent second right lower incisor.

FIG. I. THE LOWER MAXILLA.

I. Fracture at base of condyle. II. Fracture through body. III. Separation of symphysis.

On August 10th, the swelling having markedly lessened and considerable deformity being evident, chloroform was administered. By the use of fine silver wire the following teeth, which were fairly solid, were wired together; the first bicuspid and canine on the left lower jaw to the first right lower bicuspid, thus holding the separated symphysis in apposition; the first left lower to the first left upper bicuspid on one side and the second bicuspid on the right upper to the second bicuspid of the lower of the same side. The jaws were then tightly bandaged together, using the superior maxilla as a splint for the inferior. A plaster-of-Paris bandage was applied in the same way as the previous splint. There was great difficulty in holding the fragments of the ascending ramus in their proper position. Over-riding was prevented through marked pressure by means of small pads placed next the skin. After a week the wire was removed.

The bandage was kept on until after the boy's return home to

FIG. 2. THE PATIENT.

THE DRESSING IN SITU.

Syracuse, on September 9th, when Dr. F. H. Butler, of that city removed it. The condition was satisfactory and a simple bandage was reapplied. Up to this time the tube and an occasional pouring of fluid between the teeth were the means utilised for feeding.

On September 18th, all retentive apparatus was permanently removed, and the patient began to eat normally; at first, restricting his diet to soft materials. At that time the lateral motion toward the fractured side was more marked than toward the side which had

FIG. III. SHOWING IRREGULARITY OF TEETH.

FIG. IV. SHOWING APPEARANCE OF SECOND RIGHT INCISOR TOOTH.

been dislocated. Accordingly, the patient was instructed to try to gradually overcome this tendency and his efforts proved very successful.

October 22, I saw the case and found everything satisfactory, except for a considerable deposit of callus just anterior to the left tragus.

In July, 1898, I presented the case before this Society for inspection. The boy was examined at the meeting by many of the members, and but two detected that there had been anything abnormal about the inferior maxilla. I mention this fact simply to show how perfect had been the result even at that time.

FIG. V. AS PATIENT APPEARS TODAY.

A few of the teeth are irregular in position (Fig. 3), but this defect which is no more marked than in many uninjured alveola processes, could be rectified by any efficient dentist. Fortunately, the result is perfect, the lump of callus has almost disappeared and the features are unmarred.

One added point of interest is in connection with the second right incisor. (Fig. 4.)

The tooth of a well-developed healthy child of nine years of age usually belongs to the second dentition. At the injury this tooth was extracted and yet today, we have a new incisor already through the gum, to the extent of one-third of an inch; thus we may have here an example of a third dentition.

## Figures and Tables

**Fig. I. f1:**
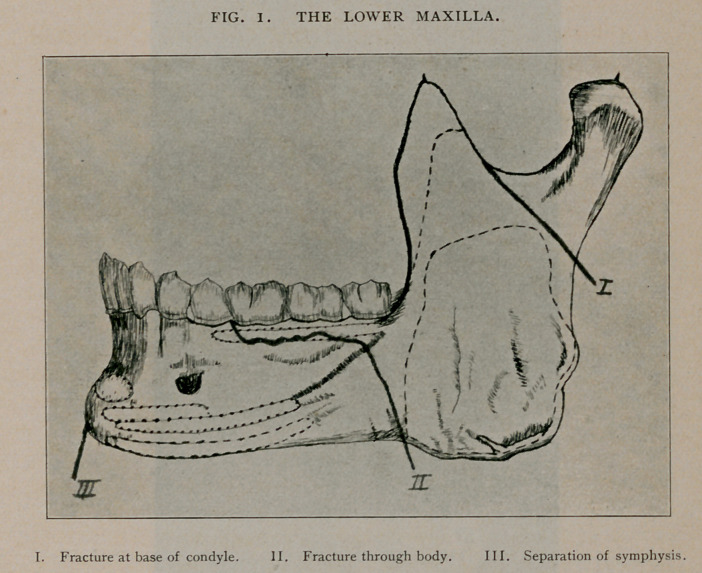


**Fig. 2. f2:**
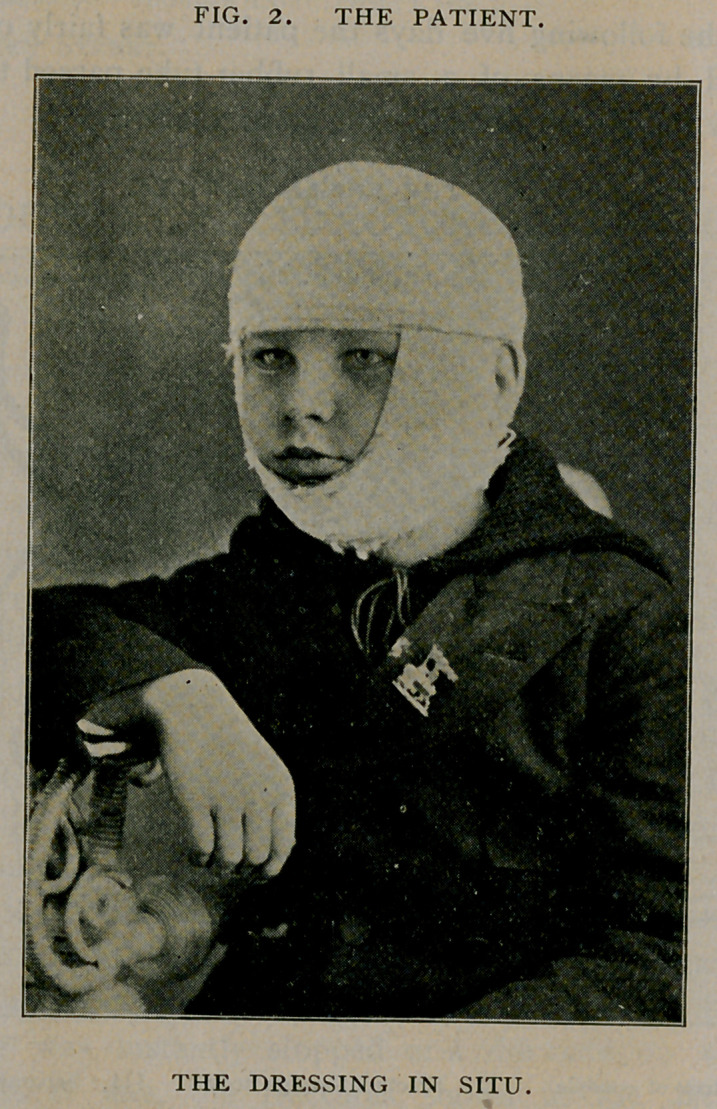


**Fig. III. f3:**
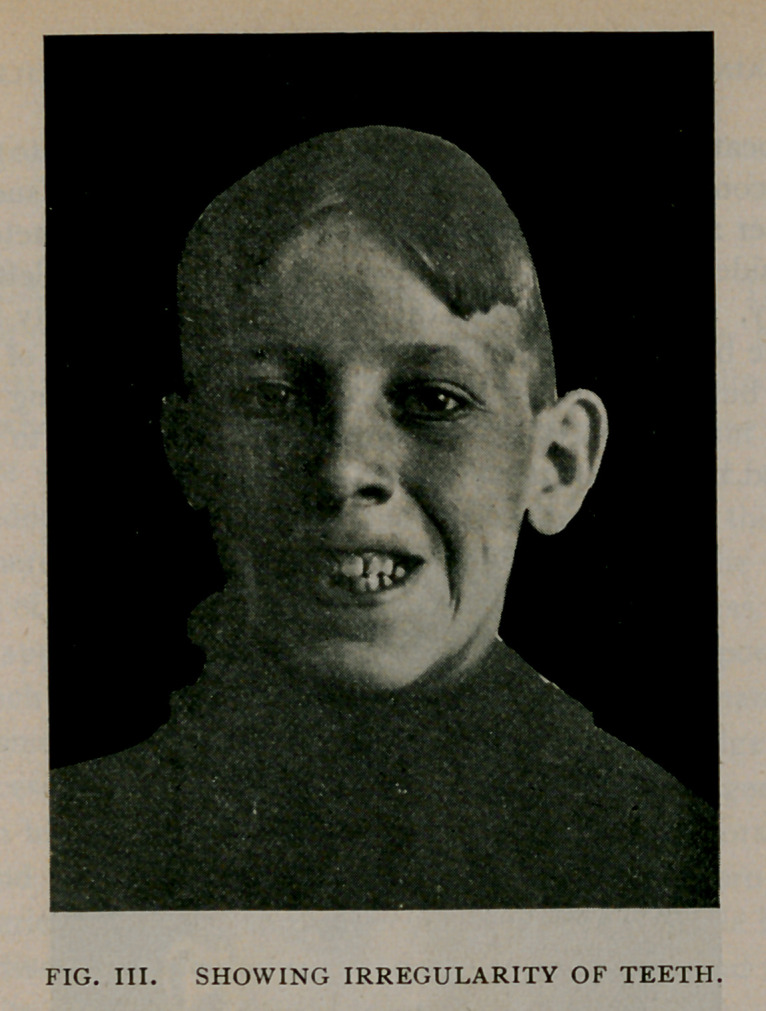


**Fig. IV. f4:**
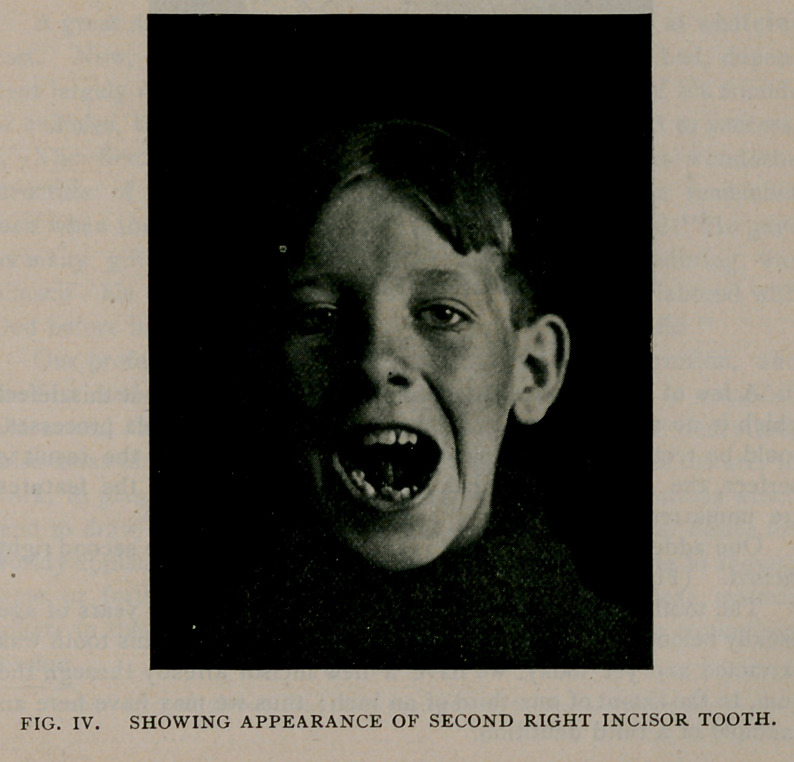


**Fig. V. f5:**